# Ventricular Assist Devices: an Evolving Field

**DOI:** 10.21470/1678-9741-2019-0043

**Published:** 2019

**Authors:** Luiz Fernando Kubrusly

**Affiliations:** 1 Incor Curitiba, Instituto Denton Cooley, Curitiba, PR, Brazil.; 2 School of Medicine, Faculdade Evangélica Mackenzie do Paraná, Curitiba, PR, Brazil.

After more than half a century of clinical development, when Dr. DeBakey in 1962 and 1966 and Dr. Cooley in 1969 first implanted devices in humans, mechanical circulatory support (MCS) has reached maturity and is now widely used for the treatment of advanced heart failure (HF).

Progress has been huge in recent years, with new challenges in candidate selection and clinical management amid the ongoing debate on the cost-effectiveness use of a finite financial healthcare resource, especially in countries like Brazil.

Circulatory support devices of the first phase of development always sought to create the systolic-diastolic pressure differential. It was inconceivable to imagine a continuous flow as a form of prolonged circulatory assistance. Thus, models such as Novacor, Electrical HeartMate and others were developed to allow patients to reach cardiac transplantation under improved clinical conditions.

Since the work of Richard Wampler and OH Frazier with Hemopump in 1988 at Texas Heart Institute, when they brought the advent of rotary blood pump technology, continuous-flow device therapy has emerged as a viable alternative in patients ineligible for cardiac transplantation.

The advances in mechanical circulatory support have shared with that of heart transplantation. Many of the decisions today in the treatment of heart failure are now focused on choosing patients for cardiac transplantation, mechanical support, or both.

Acute decompensated heart failure has emerged as a major public health problem over the last decade. It is estimated that there are 1 million hospitalizations with a primary discharge diagnosis of this syndrome annually, and that number is expected to increase substantially over the next two decades. The American Heart Association estimated that the prevalence of heart failure will increase 46% by 2030, resulting in more than 8 million people affected by the disease^[1]^.

Heart failure has become the leading cause of hospitalization in people over 65 years of age worldwide, even considering economic differences in countries. Reported death rates appear excessive both during and after hospitalization, and readmission rates reveal failure in effective long-term care. The direct costs associated with treating the 5 million Americans with chronic heart failure are mostly attributable to hospitalization. The US government estimates spending more than $26 billion a year on treating patients with heart failure. In Brazil, of all hospitalizations in the country, 21% are from some etiology of heart failure^[2,3]^.

The registry of the International Society for Heart and Lung Transplantation reports the short- and long-term outcomes of a heart transplant, with 1 and 10-year survival rates of approximately 85% and 50%, respectively. However, the number of heart transplants, both in children and in adults, had little improvement in the last few years^[4]^.

With this disproportionate number of heart transplants and the increasing number of patients with heart failure, ventricular assist devices (VADs) became the most common surgical procedure to support failing circulation, outstanding the frequency of heart transplant (Figure 1).

**Fig. 1:**
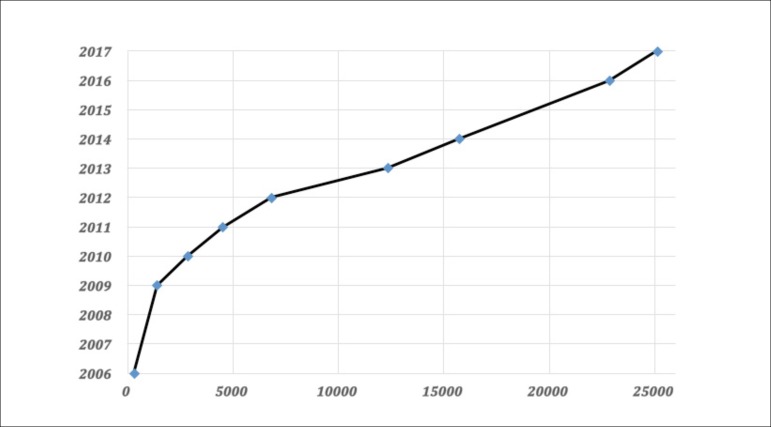
Mechanical circulatory support device implants from 2006-2017 in the United States

The shortage of donor organs enforces the establishment of waiting lists and priority allocation of patients. This shortage also encouraged the research for therapeutic alternatives, with the possibility of helping circulatory support and prompt availability when needed.

Improvements in device technology have made VADs an attractive alternative for the treatment of end-stage HF. More reliable systems have enabled bridge to transplant (BTT) therapy. The use of VADs for this group of patients is a Class IIa-C recommendation. An increasing number of patients with end-stage HF who are ineligible for a transplant can be implanted with VADs as destination therapy (DT), in order to improve survival and quality of life (Class IIa-B recommendation)^[5]^.

As mentioned, the leading indication for LVAD therapy is no longer BTT but DT in patients who are ineligible for a heart transplant. Deciding on either strategy requires an evaluation of the patient's natural course with HF *versus* the patient's chances of surviving the complications of LVAD therapy^[6]^. Durable VAD therapy in patients with end-stage HF demands a multidisciplinary approach in experienced high-volume centers, with a transplant background. There is a constant 24/7 need.

In the 1990s, a large number of patients were bridged to transplant with LVADs. The Randomized Evaluation of Mechanical Assistance for the Treatment of Congestive Heart Failure (REMATCH) trial was published in 2001 and opened the era of DT in patients with end-stage HF not eligible for a heart transplant^[7]^.

Like we said, the first-generation VADs sought to create natural circulation by producing pulsatile flow. The setup of the pneumatic chamber, driveline, controller and power source was relatively big and noisy.

Second and third-generation pumps are focused on size, biocompatibility, durability, effectiveness and infection issues. Miniaturization and improved efficiency were the main drivers of developments. The new devices were more reliable with a reduced failure rate.

The most important second-generation VAD, the redesigned HeartMate II (Abbott, St. Paul, MN, USA), proved successful as a BTT device in a prospective multicenter study published in 2007. Patients with end-stage HF improved after implantation of this device in terms of functional status and quality of life. The FDA approved the HeartMate II as a BTT in 2008 and a DT in 2010. Clinical results after implantation of the HeartMate II improved steadily to 85% 1-year survival^[8]^. Patients with this continuous-flow device demonstrated dramatically improved survival compared with patients on first-generation pulsatile flow devices.

Third-generation LVADs generate continuous blood flow through a centrifugal pump design. The first relevant third-generation LVAD is the HeartWare Ventricular Assist Device (HVAD(r)) (Medtronic, Minneapolis, MN, USA), which allows intrapericardial and less invasive implantation. The ADVANCE (HeartWare Left Ventricular Assist Device for the Treatment of Advanced Heart Failure) trial^[9]^ reported 86% 1-year survival after HVAD implantation, with significant improvement in functional capacity and quality of life. Based on this BTT evaluation, the HVAD(r) received FDA approval in 2012.

The HeartMate III is the latest third-generation LVAD, a centrifugal continuous-flow LVAD with a fully magnetically levitated impeller. The MOMENTUM 3 (Multicentre Study of MagLev Technology in Patients Undergoing Mechanical Circulatory Support Therapy with HeartMate 3) study compared the HeartMate III with the axial flow pump HeartMate II; the follow-up data are promising. The important finding was that none of the patients with HeartMate III experienced a pump thrombosis^[10]^. This complication is probably restrained by the design of the pump, which is characterized by the relatively large housing of the impeller and by the intermittent creation of at least some 'pulsatility' by the automated rotational speed variation. The implantation techniques for the third-generation centrifugal CF pumps preserve the pericardial integrity. Whether this advance will help prevent short-term and long-term right HF is still unknown.

## COMPLICATIONS

RIGHT VENTRICULAR FAILURE is a major factor for the mortality rate among patients with an LVAD, particularly in patients who are at Intermacs levels 1 and 2 at the time of implantation. The risk of death after LVAD implantation due to RV failure is highest in the early postoperative period. Late-onset right ventricular failure contributes to morbidity and mortality after initially successful LVAD implantation.

PUMP THROMBOSIS is a serious complication requiring either surgical pump exchange or systemic thrombolysis. Although each of these options is technically feasible, each one results in a large reduction in the 1-year survival rate compared to that with a primary implant. The obvious increase in LVAD thrombosis may be explained by the significantly longer support duration with centrifugal devices. Adherence to standard anticoagulation recommendations can result in a reduction in the risk of pump thrombosis.

BLEEDING COMPLICATIONS, mainly gastrointestinal, are a great risk of death after VAD implantation. The occurrence of major bleeding may approximate 23%, with a recurrence of nearly 10%. It remains a question if and how the long-term continuous flow contributed to the development of arteriovenous malformations and bleeding events.

DEVICE-RELATED INFECTIONS remain a common cause of morbidity and mortality in patients with VADs. Transcutaneous drivelines obviously facilitate ascending staphylococci-dominated infections. The incidence of device infections varies between 13% and 80%. There is data today supporting the decrease of this complication with recent devices.

NEUROLOGICAL COMPLICATIONS represents the most devastating risk of death in the mid- to long-term after LVAD implantation. This risk stays constant throughout the first 4 years after LVAD implantation. Previous cerebrovascular accident, hyponatremia, low albumin levels, elevated right atrial pressure, enlarged right ventricular end-diastolic dimensions, atrial fibrillation, postoperative infection and supratherapeutic anticoagulation levels correlate with the incidence of cerebrovascular accident.

DEVICE MALFUNCTION 50% of patients with a VAD experience device malfunctions other than pump thrombosis within 1 year postoperatively. The durability and functionality of LVADs are influenced by numerous factors, including implantation technique, anatomical constraints and complications such as infection and bleeding, anticoagulation, pump settings and device design. Centrifugal pump devices have demonstrated improved durability compared with pulsatile devices in studies with up to 24 months of follow-up.

## PERSPECTIVES

Transcutaneous energy transfer system and better biocompatibility could probably be the most important factors in the near future for VADs. This kind of advances will be capable of yielding better quality of life with reduced risk of complications for this population of patients.
